# Supplementary figures and images for: The mechanisms behind perivascular fluid flow
Source: PLoS One. 2020 Dec 29;15(12):e0244442. doi: 10.1371/journal.pone.0244442 (PMC7771676; doi:10.1371/journal.pone.0244442)

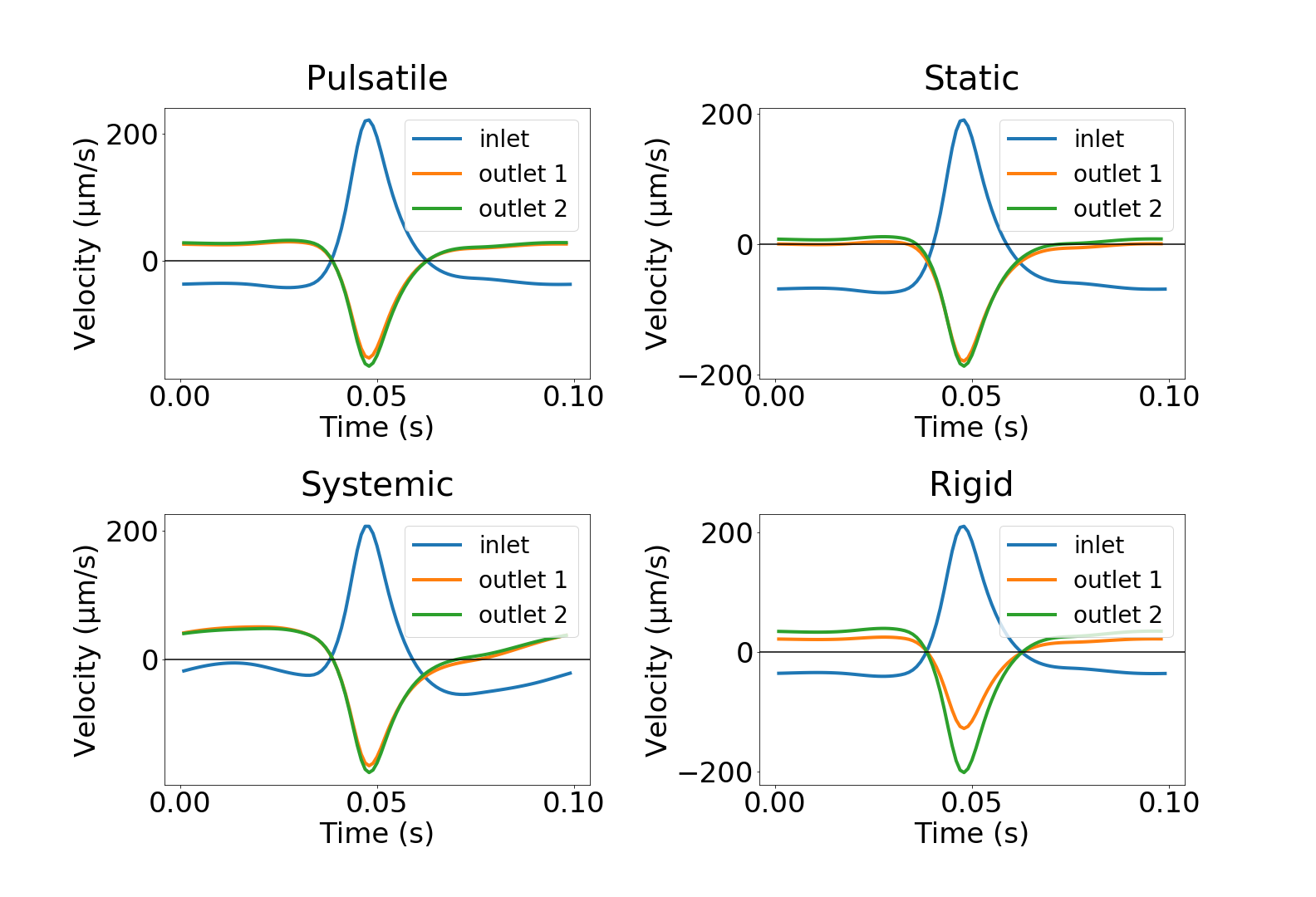

Supplement: S1 Fig — Cardiac frequency: 10 Hz. The outward pointing normal at the inlet defines the positive direction; negative values thus refer to flow downwards (into the PVS at the inlet, and out of the PVS at the outlet). All models predict bi-directional flow during systole, with fluid leaving the domain at both inlet and outlets. The peak velocity amplitude is slightly higher at the inlet during systole mainly due to the smaller area for flow compared to the combined area at the outlets. The velocity during diastole differ more between the models, and more (in absolute value) between at the inlet and outlet. (TIF) [file pone.0244442.s004.tif]

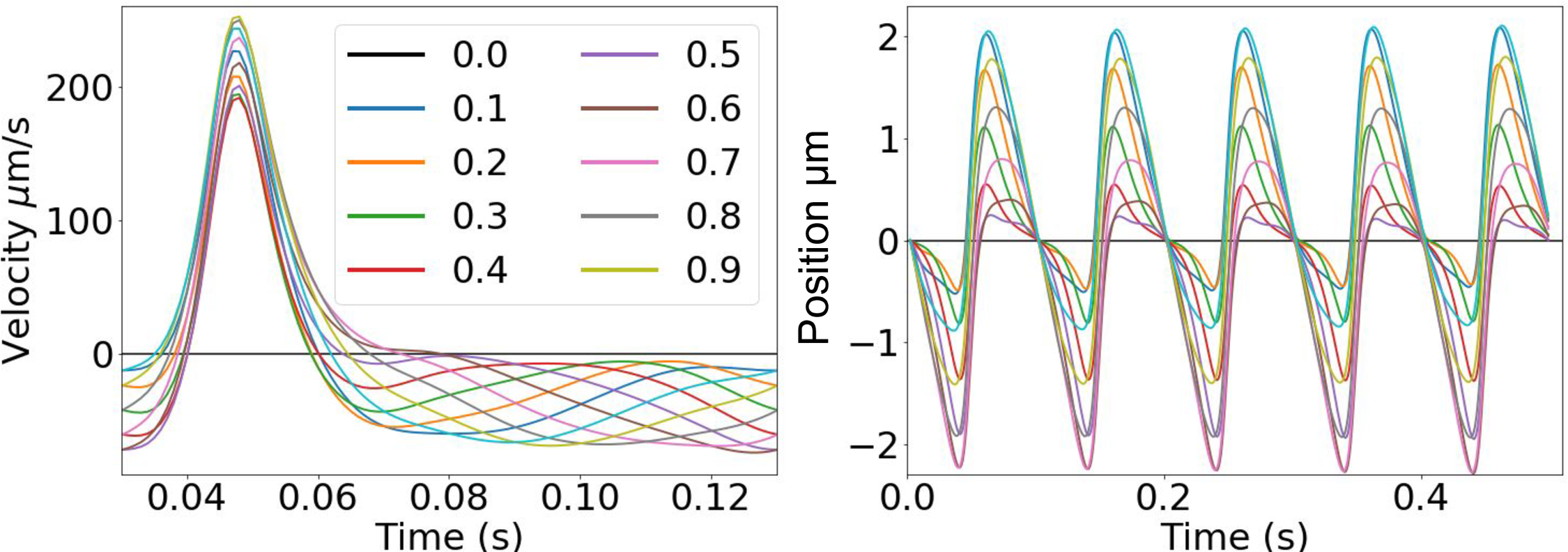

Supplement: S2 Fig — The shift θ was represented by different fractions of the cardiac cycle ranging from 0 to 0.9 of steps 0.1. The net flow velocity was lower than 0.5 μm/s for all shifts θ. (TIF) [file pone.0244442.s005.tif]

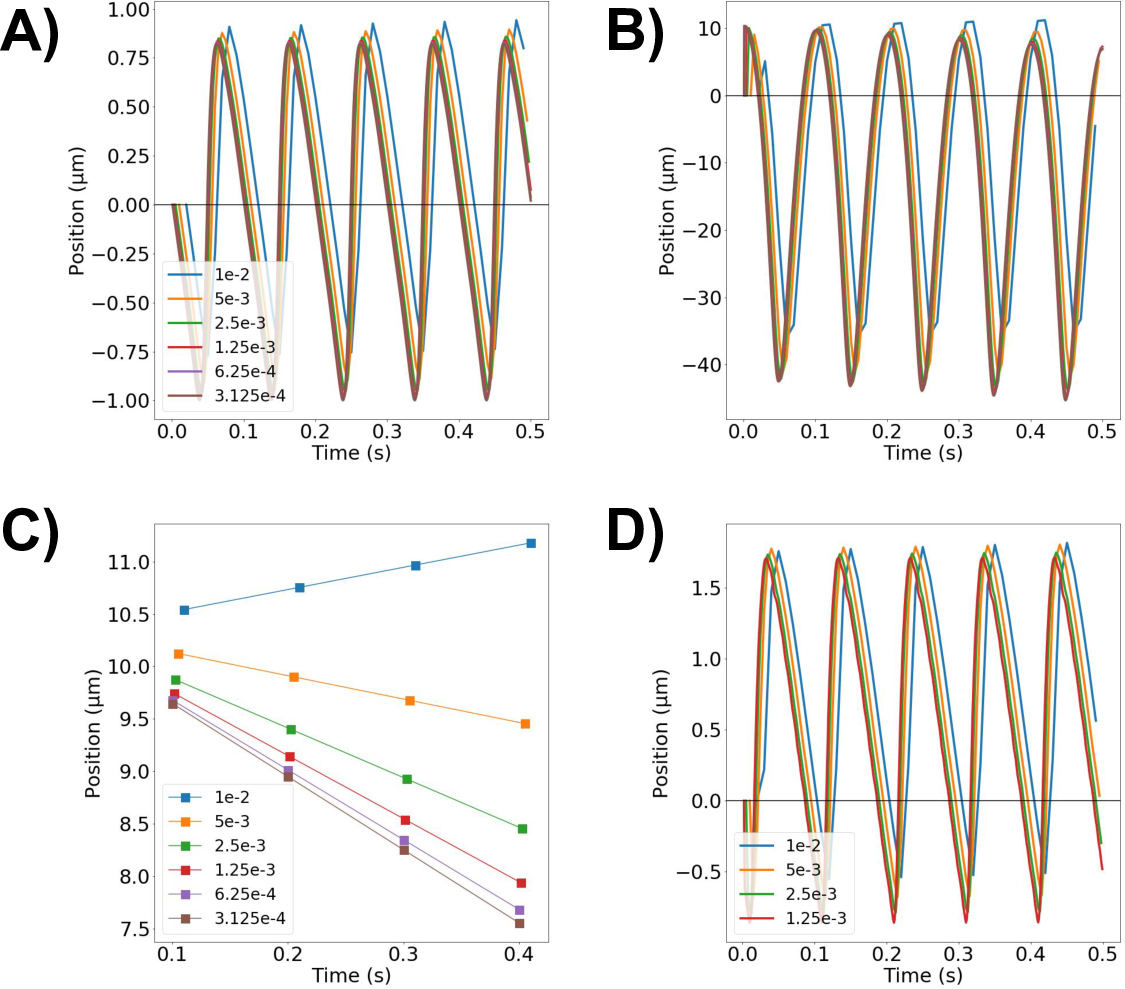

Supplement: S3 Fig — Position over time for the idealized models of length 1 mm (A) and 100 mm (B) for different time resolutions (Δt). Key output quantities converge as the time resolution is reduced as expected. For a PVS length of 100 mm, the time step required for convergence was lower than for the shorter models. C) Peaks of the position over time for different time resolutions (L = 100 mm). We note that the computed net flow velocity strongly depends on the time resolution, but that a time step of 1 ms is sufficient. D) Position over time for the bifurcating arterial geometry (C0075) for different time resolutions. The time step of 1 ms is again sufficient. (TIF) [file pone.0244442.s006.tif]

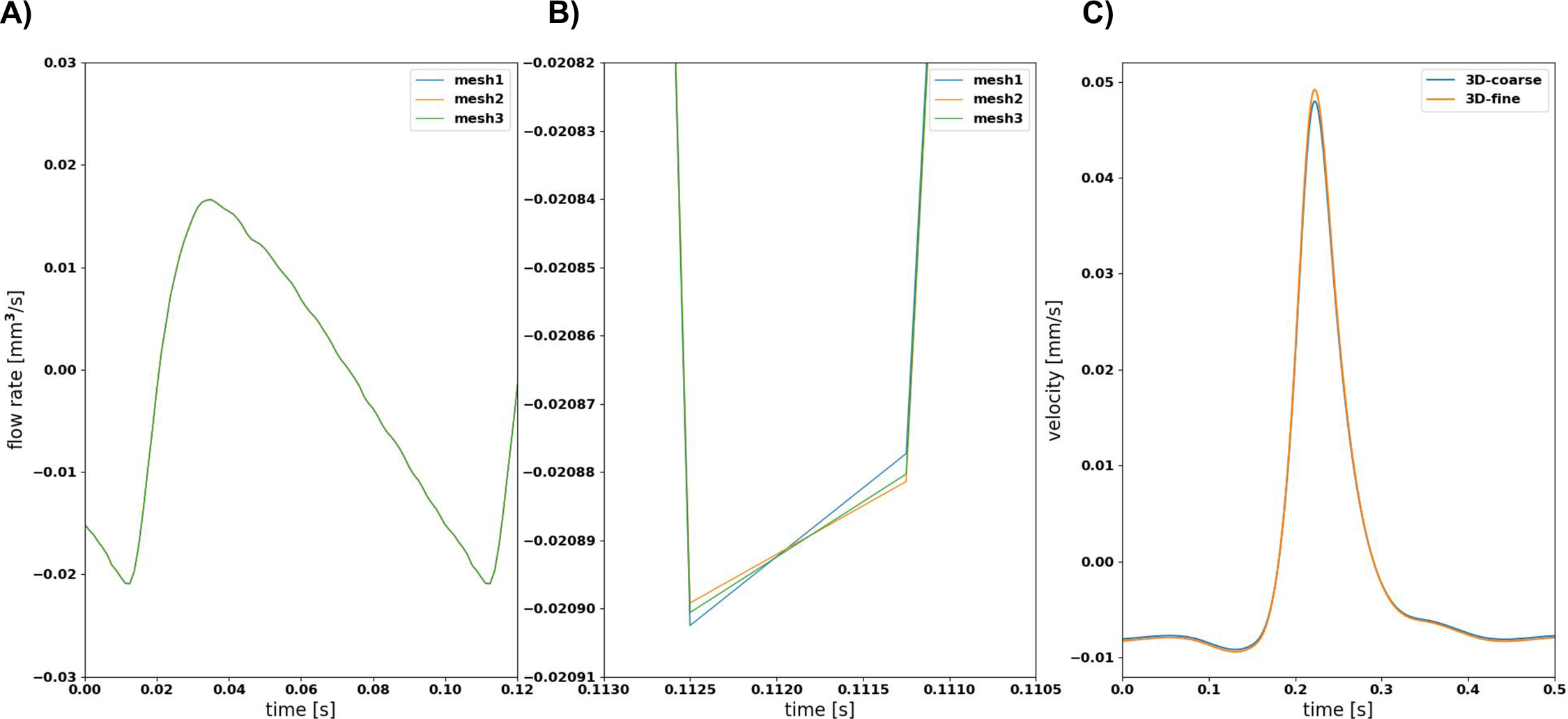

Supplement: S4 Fig — A) Flow rate in the 2D model with different mesh resolutions. B) Close-up for a very short time period in A) to emphasize the small differences between the different mesh resolutions in 2D. C) Very small differences were also observed in 3D where two meshes were tested. Peak velocity obtained in the two meshes differed by ≈ 2.5%. (TIF) [file pone.0244442.s007.tif]
